# The Synthesis and Evaluations of the ^**68**^Ga-Lissamine Rhodamine B (LRB) as a New Radiotracer for Imaging Tumors by Positron Emission Tomography

**DOI:** 10.1155/2016/8549635

**Published:** 2016-02-02

**Authors:** Xuena Li, Yafu Yin, Bulin Du, Na Li, Yaming Li

**Affiliations:** Department of Nuclear Medicine, The First Hospital of China Medical University, Shenyang 110001, China

## Abstract

*Purpose*. The aim of this study is to synthesize and evaluate ^68^Ga-labeled Lissamine Rhodamine B (LRB) as a new radiotracer for imaging MDA-MB-231 and MCF-7 cells induced tumor mice by positron emission tomography (PET).* Methods*. Firstly, we performed the radio synthesis and microPET imaging of ^68^Ga(DOTA-LRB) in athymic nude mice bearing MDA-MB-231 and MCF-7 human breast cancer xenografts. Additionally, the evaluations of ^18^F-fluorodeoxyglucose (FDG), as a glucose metabolism radiotracer for imaging tumors in the same xenografts, have been conducted as a comparison.* Results*. The radiochemical purity of ^68^Ga(DOTA-LRB) was >95%. MicroPET dynamic imaging revealed that the uptake of ^68^Ga(DOTA-LRB) was mainly in normal organs, such as kidney, heart, liver, and brain and mainly excreted from kidney. The MDA-MB-231 and MCF-7 tumors were not clearly visible in PET images at 5, 15, 30, 40, 50, and 60 min after injection of ^68^Ga(DOTA-LRB). The tumor uptake values of ^18^F-FDG were 3.79 ± 0.57 and 1.93 ± 0.48%ID/g in MDA-MB-231 and MCF-7 tumor xenografts, respectively.* Conclusions*. ^68^Ga(DOTA-LRB) can be easily synthesized with high radiochemical purity and stability; however, it may be not an ideal PET radiotracer for imaging of MDR-positive tumors.

## 1. Introduction

Tumor growth depends on the energy metabolism of the supply, and the biological energy of tumor has received much attention in recent years [1, 2]. A metabolic shift from oxidative phosphorylation in the mitochondria to glycolysis in cancer was first described about 80 years ago by Warburg [3]. Increased glucose metabolism is an important feature of cancer [4]. Active glucose uptake by cancer cells constitutes the basis for ^18^F-fluorodeoxyglucose-positron emission tomography (^18^F-FDG PET), an imaging technology commonly used in cancer diagnosis. However, the reverse Warburg effect was recently found in a human breast cancer model [5–7]. The researchers found that breast cancer cells showed a significant increase activity in mitochondria [8]. However, the development of molecular imaging probes targeting tumor mitochondria is very limited.

It has been reported that the mitochondrial potential in carcinoma cells is significantly higher than that in normal epithelial cells [9, 10], and mitochondrial potential is negative; many organic cations are driven through these cell membranes and able to localize in the mitochondria of tumor cells [11–13]. Several studies proposed to use the ^64^Cu(DO3A-xy-TPEP) and ^18^F-labeled phosphonium cations as PET radiotracers for tumor mitochondria, but they had high background in normal organs [14, 15]. Lissamine Rhodamine B (LRB) is a derivative of rhodamine, which has been used as probe for mitochondrial potentials. ^64^Cu-LRB, a radiotracer targeting tumor mitochondria for U87MG human glioma xenografts, has low radioactivity accumulation in the brain, and ^64^Cu requires high energy cyclotron for production, both of which limit the clinical application in the tumor [16]. ^68^Ga is a generator-produced radionuclide, and its half-life is 67.6 min, which is produced by ^68^Ge/^68^Ga generator; the production of ^68^Ga is not dependent on the cyclotron.

**Figure 1:**
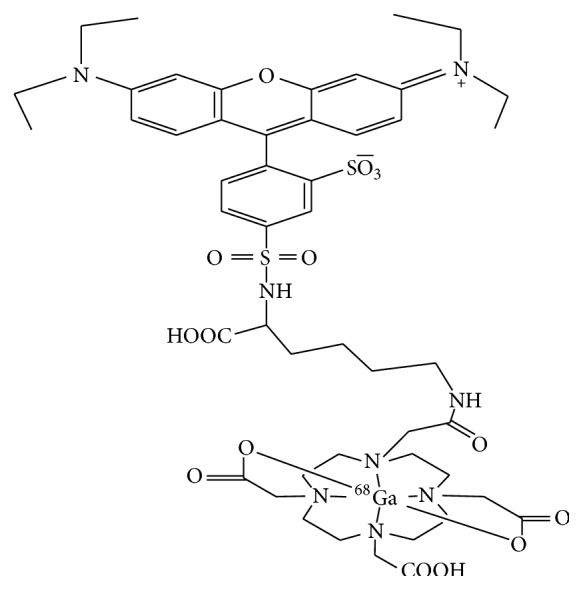
Proposed structure of ^68^Ga(DOTA-LRB).

The objective of our study is to synthesize and evaluate ^68^Ga-labeled Lissamine Rhodamine B (LRB) (Figure 1) as a new radiotracer for imaging MDA-MB-231 and MCF-7 cells induced tumor mice by positron emission tomography (PET). Additionally, ^18^F-FDG, as a glucose metabolism radiotracer for imaging tumors in the same xenografts, was further evaluated as a comparison.

## 2. Materials and Methods

2-(6-(Diethylamino)-3-(diethyliminio)-3H-xanthen-9-yl)-5-(N-(2-(2-(4,7,10-tris(carboxymethyl)-1,4,7,10-tetraazacyclododecan-1-yl)acetamido)ethyl)sulfamoyl)-benzenesulfonate (DOTA-LRB) was kindly provided by Dr. Shuang Liu (School of Health Sciences, Purdue University, West Lafayette, Indiana, USA), and the method of synthesis and purification was described in the previous study [16].

### 2.1. HPLC Methods

The semiprep HPLC method used a Waters 2545+BIOSCAN Flowcount system equipped with a UV/Vis detector (*λ* = 254 nm) and CHROM-MATRIX C-18 semiprep column (10 mm × 250 mm). The flow rate was 3 mL/min. The mobile phase was isocratic with 70% A (0.1% TFA in water) and 30% B (0.1% TFA in methanol) at 0–5 min, followed by a gradient mobile phase going from 30% B at 5 min to 80% B at 20 min, followed by a gradient mobile phase going from 80% B at 20 min to 30% B at 25 min. The radio-HPLC analysis method used a system (Waters, Inc., USA) consisting of Agilent TC-18 Chromatographic column (4.6 × 250 mm, 5 *μ*m), Perkinzimer online radioactivity detector, and a UV detector (*λ* = 254 nm). The flow rate was 1 mL/min. The mobile phase was isocratic with 60% A (0.1% TFA in water) and 40% B (0.1% TFA in methanol) at 0-1 min, followed by a gradient mobile phase going from 40% B at 1 min to 90% B at 40 min, followed by a gradient mobile phase going from 90% B at 40 min to 98% B at 45 min.

### 2.2. ^68^Ga Radiolabeling

^68^Ga was obtained from a ^68^Ge/^68^Ga generator (Garching GmbH, Germany) eluted with 0.1 N HCl. Fresh ^68^Ga was loaded into an ion exchange column. By using a mixture of 400 *μ*L 97.6% acetone and 0.05 M hydrochloric acid, ^68^Ga was eluted from the exchange column and added to the solution containing 10 *μ*g DOTA-LRB in 400 *μ*L 0.25 M HEPES (pH 4.0); the reaction mixture was then heated at 100°C for 20 min.

### 2.3. Cancer Cell Line, Nude Mice, and Cancer Models

The human breast cancer MDA-MB-231 and MCF-7, purchased from Shanghai Cell Bank of Chinese Academy of Sciences, were used in our experiments and preparation of animal models. The human breast cancer MDA-MB-231 and MCF-7 cells were maintained in DMEM (Dulbecco's modified Eagle's medium) (GIBCO, Inc.) supplemented with 10% fetal bovine serum (GIBCO, Inc.) with 100 units/mL streptomycin and 100 units/mL penicillin. Cells were grown in a humidified atmosphere at 37°C with 5% carbon dioxide.

All experiments were performed using 6-week-old female athymic nude mice purchased from Shanghai Silaike Experimental Animal Co. Ltd. Athymic nude mice derived are in compliance with regulations of our institution. All animal experiments were approved by the China Medical University Animal Care and Use Committee.

Subcutaneous injection of 5 × 10^6^ tumor cells into the breast fat pad of female athymic nude mice generated the tumor model. When the tumor volume was 100~300 mm^3^ (about 3~4 weeks after inoculation), the mice underwent small animal PET imaging studies.

### 2.4. MicroPET Imaging

#### 2.4.1. ^68^Ga(DOTA-LRB) MicroPET Imaging and ^18^F-FDG MicroPET Imaging

The tumor-bearing MDA-MB-231 (*n* = 6) and MCF-7 (*n* = 6) nude mice were imaged in the Inveon microPET scanner (Siemens Medical Solutions). Animals were anesthetized by isoflurane. Each tumor-bearing mouse was injected with ~100 *μ*Ci of ^68^Ga(DOTA-LRB) via the tail vein; 10 min static scans were obtained at 5, 15, 30, 40, 50, and 60 min p.i. Each tumor-bearing mouse was injected with ~100 *μ*Ci of ^18^F-FDG via the tail vein; 10 min scans were acquired at 1 h after injection. The all images were reconstructed by a 3D-OSEM (three-dimensional ordered subsets expectation maximum) algorithm. The boundary was determined with the threshold of 50%. The radioactivity concentration of the tumor or normal organ was obtained from uptake values within the ROI [17].

### 2.5. Statistical Analysis

Quantitative data is expressed as mean ± SD. Means were compared using Student's *t*-test. *P* < 0.05 was considered statistically significant.

## 3. Results

### 3.1. Chemistry and Radiochemistry

**Figure 2:**
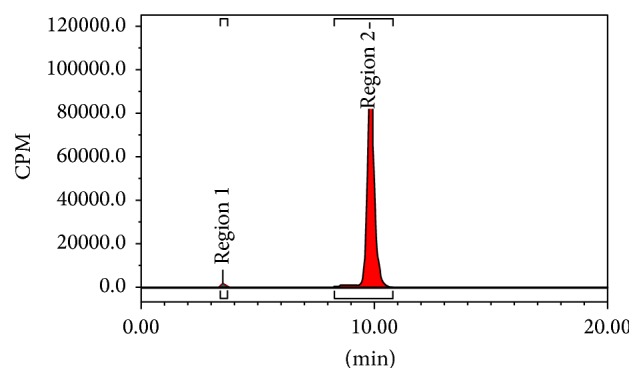
Radio-HPLC chromatogram of ^68^Ga(DOTA-LRB).

**Figure 3:**
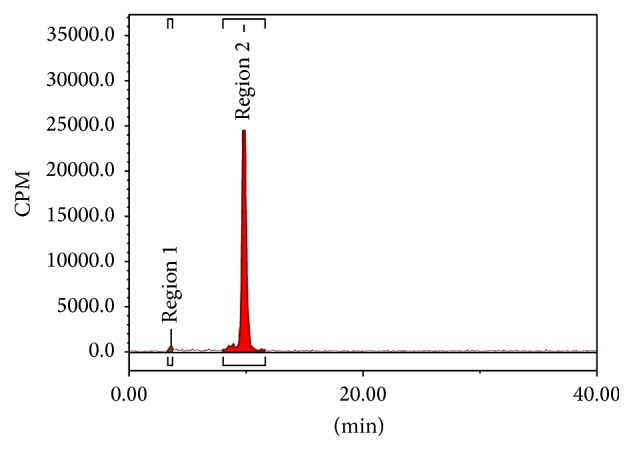
Radio-HPLC chromatogram of ^68^Ga(DOTA-LRB) in PBS at 37°C for 2 h.

The retention time of ^68^Ga(DOTA-LRB) was 9.8 min. The radiochemical purity of final product was 98.9% (Figure 2); it was analyzed by an analytical HPLC. The experiments in vitro demonstrated that radiochemical purity of ^68^Ga(DOTA-LRB) was >95% in PBS at 37°C for 2 h (Figure 3).

### 3.2. ^68^Ga(DOTA-LRB) MicroPET Imaging

**Figure 4:**
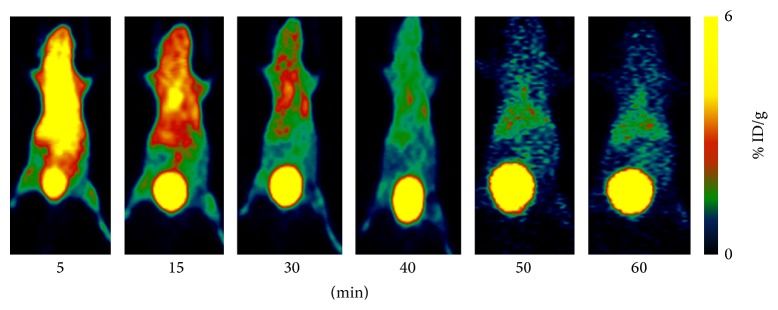
Whole-body coronal microPET images of MDA-MB-231 tumor-bearing mouse at 5, 15, 30, 40, 50, and 60 min after injection of ~100 *μ*Ci ^68^Ga(DOTA-LRB).

Figure 4 showed microPET images of MDA-MB-231 breast cancer-bearing mouse administered ~100 *μ*Ci of ^68^Ga(DOTA-LRB) at 5, 15, 30, 40, 50, and 60 min p.i. The MDA-MB-231 tumors were not clearly visible with high contrast at all the time points examined for ^68^Ga(DOTA-LRB) PET imaging.

**Figure 5:**
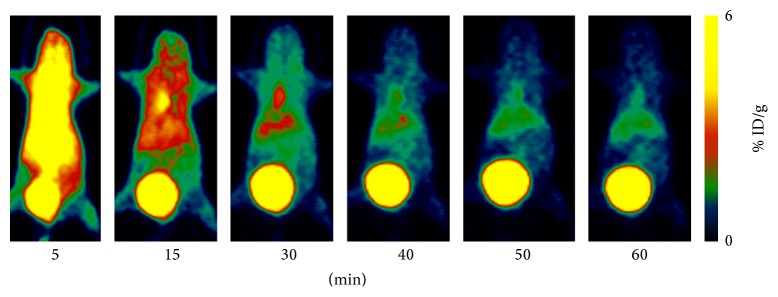
Whole-body coronal microPET images of tumor-bearing MCF-7 mouse at 5, 15, 30, 40, 50, and 60 min after injection of ~100 *μ*Ci ^68^Ga(DOTA-LRB).

Figure 5 showed microPET images of MCF-7 breast cancer-bearing mice administered ~100 *μ*Ci of ^68^Ga(DOTA-LRB) at 5, 15, 30, 40, 50, and 60 min p.i. The uptake of ^68^Ga-labeled LRB was negative at all the time points.

MicroPET dynamic imaging revealed the uptake of ^68^Ga(DOTA-LRB) in normal organs (kidney, heart, and liver) and the excretion from the kidney. It had very low ^68^Ga(DOTA-LRB) radioactivity accumulation in the brain. The uptakes of ^68^Ga(DOTA-LRB) in kidneys, liver, heart, and brain were 4.44 ± 2.32, 2.11 ± 0.98, 2.17 ± 0.90, and 0.53 ± 0.19% ID/g at 30 min p.i., respectively.

### 3.3. ^18^F-FDG MicroPET Imaging

**Figure 6:**
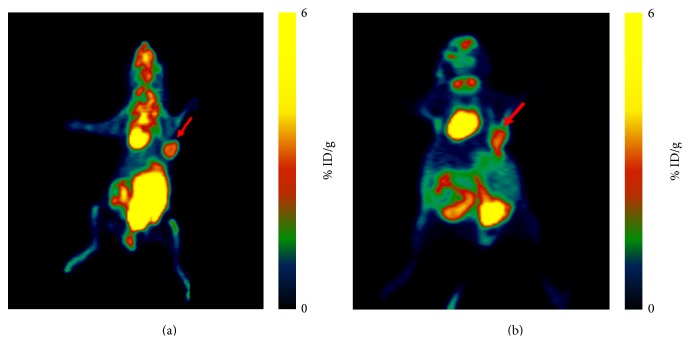
(a) Whole-body coronal microPET image of tumor-bearing MDA-MB-231 mouse at 60 min after injection of ~100 *μ*Ci ^18^F-FDG. Tumors are indicated by arrows. (b) Whole-body coronal microPET image of a tumor-bearing MCF-7 mouse at 60 min after injection of ~100 *μ*Ci ^18^F-FDG. Tumors are indicated by arrows.

Figure 6 showed microPET images of MDA-MB-231 breast cancer-bearing mouse and MCF-7 breast cancer-bearing mouse administered ~100 *μ*Ci of ^18^F-FDG at 60 min p.i. The tumor uptake values were 3.79 ± 0.57 and 1.93 ± 0.48 %ID/g in MDA-MB-231 and MCF-7 breast cancer-bearing mice, respectively. The tumor uptake of ^18^F-FDG was visually higher than that of ^68^Ga(DOTA-LRB).

## 4. Discussion

Increase of mitochondrial transmembrane potential (ΔΨ*m*) is an important characteristic of cancer [18–20]. Molecular imaging probes based on mitochondrial transmembrane potential have attracted intensive research attention in recent years. Although many radiolabeled cationic tracers have been reported, they all need to be produced by the cyclotron. ^68^Ga is produced by ^68^Ge-^68^Ga generator. ^68^Ga is the short half-life radionuclide, which is difficult for commercial distribution. The major advantage of the generator is that it can produce continuous source of ^68^Ga independent of the cyclotron; ^68^Ga-labeled biomolecules have great advantages in clinical application [21–23].

This is the first synthesis study for ^68^Ga(DOTA-LRB), which was easily labeled with ^68^Ga and the radiochemical purity of ^68^Ga(DOTA-LRB) could reach more than 95% with HPLC purification. The HPLC retention time was 9.8 min. The experiments in vitro demonstrated that ^68^Ga(DOTA-LRB) was stable in PBS at 37°C for 2 h.

MicroPET dynamic imaging revealed that normal organs (kidney, heart, and liver) had ^68^Ga(DOTA-LRB) uptake and mainly excreted from the kidney. It had very low ^68^Ga(DOTA-LRB) radioactivity accumulation in the normal brain tissue. The distribution of ^68^Ga(DOTA-LRB) in normal tissues was consistent with that of ^64^Cu(DOTA-LRB) [16]. ^68^Ga(DOTA-LRB) was very low accumulation in the normal brain; it is probably because this compound is not able to cross the blood brain barrier (BBB). ^68^Ga(DOTA-LRB) showed better biodistribution in normal organs in this study, compared with another report using ^64^Cu-labeled acridinium cation, which is high and prolonged liver uptake [24].

The previous study showed that the uptake of ^64^Cu(DOTA-LRB) was positive in U87MG human glioma xenografts [16], whereas our study showed ^68^Ga(DOTA-LRB) uptake in MDA-MB-231 and MCF-7 breast cancer cells was negative. We attributed the difference to different cell lines. The study by Dr. Liu's group with ^64^Cu(DOTA-LRB) used the U87MG human glioma cell, which is negative expression of multidrug resistance (MDR) protein tumor cell [16], whereas our study used the MDA-MB-231 and MCF-7 breast cancer cell lines, which are not MRP-negative cancer cell. It was reported that the MDR had positive expression in MDA-MB-231 and MCF-7 breast cancer cells [25]. Because some cations are the substrate for MDR protein, cationic radiotracers have been clinically used for noninvasive monitoring of the multidrug resistance transport function in tumors [26, 27]. Lissamine Rhodamine B (LRB) is a member of rhodamine derivatives, which is also the substrate for MDR protein. Therefore, lower ^68^Ga(DOTA-LRB) tumor uptake in the two breast cancer cells may be associated with MDR. ^68^Ga(DOTA-LRB) may enter the tumor cells but pump out of the tumor cells as a substrate for MDR. These results suggested that the ^68^Ga(DOTA-LRB) molecular probe may be used to measure the MDR of tumor.

We also found that the uptake of MDA-MB-231 and MCF-7 was positive by ^18^F-FDG microPET imaging, and the uptake of MDA-MB-231 in the high invasive ^18^F-FDG tumor was slightly higher than that in the low invasive MCF-7 tumor, but without statistical significance. Previous group has demonstrated that some types of aggressive breast cancers are associated with a high uptake for ^18^F-FDG, while more indolent breast cancers are characterized by low ^18^F-FDG uptake [28, 29].

In non-MDR negative tumors, the uptake of ^68^Ga(DOTA-LRB) was low in MDA-MB-231 xenografts and MCF-7 xenografts, but it was very easy to synthesize. In the future study, we will perform a study of ^68^Ga(DOTA-LRB) in MDR negative tumors.

## 5. Conclusions

^68^Ga(DOTA-LRB) can be easily synthesized with high radiochemical purity and stability. ^68^Ga(DOTA-LRB) may be not an ideal PET radiotracer for tumor imaging of non-MDR-negative tumors.
